# Asymmetry between right and left fundus images identified using convolutional neural networks

**DOI:** 10.1038/s41598-021-04323-3

**Published:** 2022-01-27

**Authors:** Tae Seen Kang, Bum Jun Kim, Ki Yup Nam, Seongjin Lee, Kyonghoon Kim, Woong-sub Lee, Jinhyun Kim, Yong Seop Han

**Affiliations:** 1 Department of Ophthalmology, Gyeongsang National University Changwon Hospital, #11 Samjeongja-ro, Seongsan-gu, Changwon, 51472 Republic of Korea; 2 Department of Ophthalmology, Chungnam National University Sejong Hospital, Sejong, Republic of Korea; 3 Department of AI Convergence Engineering, Gyeongsang National University, Jinju, Republic of Korea; 4 School of Computer Science & Engineering, Kyungpook National University, Daegu, Republic of Korea; 5 Department of Information and Communication Engineering, Gyeongsang National University, Tongyeong, Republic of Korea; 6 Department of Ophthalmology, Institute of Health Sciences, Gyeongsang National University College of Medicine, Jinju, Republic of Korea

**Keywords:** Anatomy, Medical research, Computational models

## Abstract

We analyzed fundus images to identify whether convolutional neural networks (CNNs) can discriminate between right and left fundus images. We gathered 98,038 fundus photographs from the Gyeongsang National University Changwon Hospital, South Korea, and augmented these with the Ocular Disease Intelligent Recognition dataset. We created eight combinations of image sets to train CNNs. Class activation mapping was used to identify the discriminative image regions used by the CNNs. CNNs identified right and left fundus images with high accuracy (more than 99.3% in the Gyeongsang National University Changwon Hospital dataset and 91.1% in the Ocular Disease Intelligent Recognition dataset) regardless of whether the images were flipped horizontally. The depth and complexity of the CNN affected the accuracy (DenseNet121: 99.91%, ResNet50: 99.86%, and VGG19: 99.37%). DenseNet121 did not discriminate images composed of only left eyes (55.1%, p = 0.548). Class activation mapping identified the macula as the discriminative region used by the CNNs. Several previous studies used the flipping method to augment data in fundus photographs. However, such photographs are distinct from non-flipped images. This asymmetry could result in undesired bias in machine learning. Therefore, when developing a CNN with fundus photographs, care should be taken when applying data augmentation with flipping.

## Introduction

In the mid-twentieth century, Hubel and Wiesel^1^ identified simple and complex cells in the visual cortex of cats. Simple cells respond to visual angles and complex cells are connected to the simple cells. In 1980, Fukushima^2^ stated that the visual cortex was composed of multiple layers of simple and complex cells.

The multi-layered structure of the visual cortex inspired the creation of convolutional neural networks (CNNs)^3^. Different parts of images pass through the corresponding areas in different convolutional layers of CNNs. Kernels extract and learn features from the image as it passes through. This multi-layered connection in CNNs is similar to the animal visual cortex, so animal neuron activity can be predicted using CNNs^4^. Sometimes, CNNs have shown remarkable capabilities in identifying features of images that human vision cannot (for example, smoking status from retinal images^5^).

The accuracy of CNNs in diagnosing ophthalmic diseases using fundus images has been evaluated in several studies^6^. Some such studies used image-flipping to augment data^7,8^. Because researchers thought that left and right fundus images were mirror-symmetric, they believed that flipped images corresponded to actual ones. However, if the fundus photographs of both eyes are not mirror-symmetric, then CNNs could learn unexpected features from flipped images. Thus, the flipping method could cause undesired bias.

**Figure 1:**
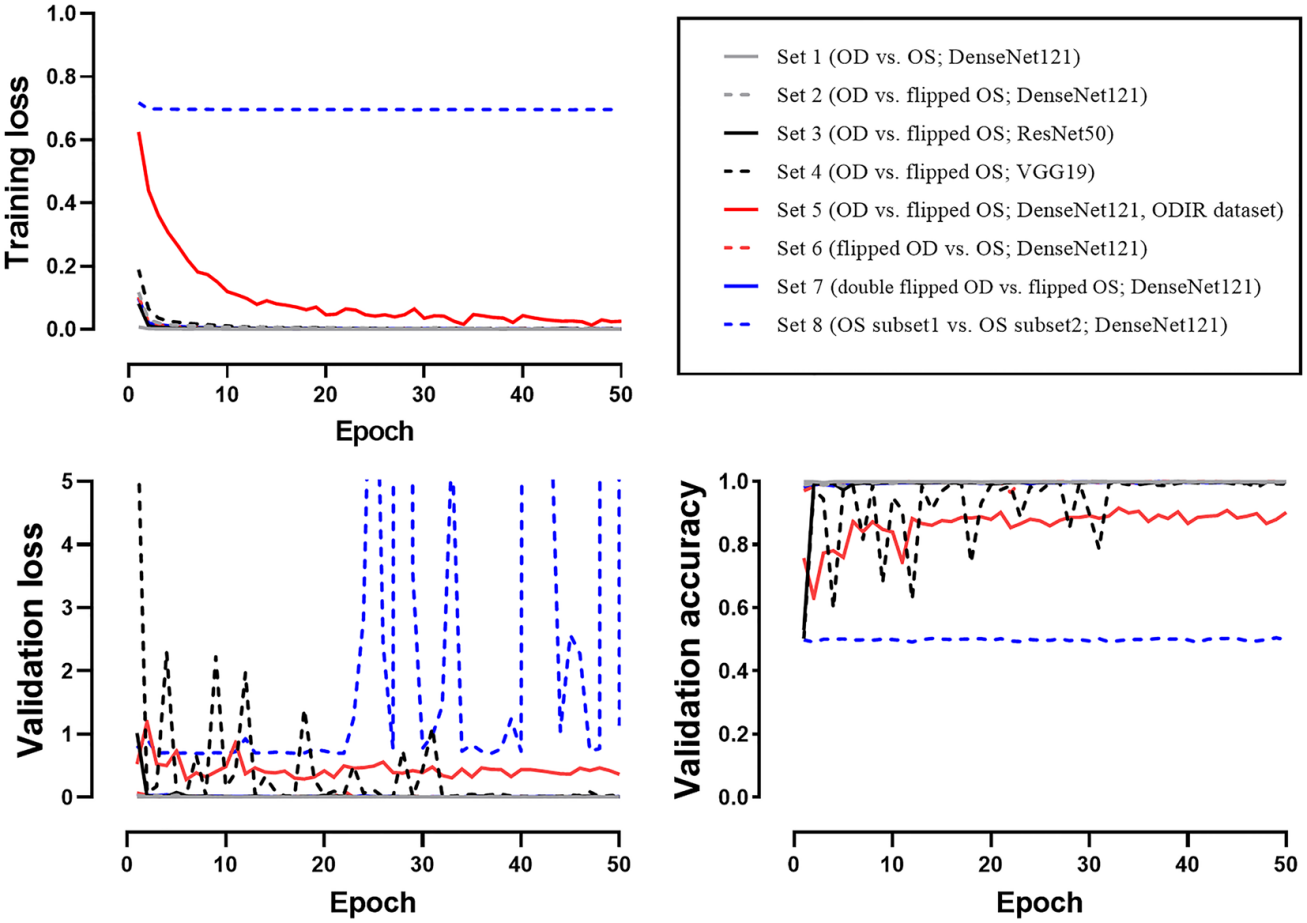
Training and validation plot. The training and validation losses approached zero for Sets 1–7, while the losses of Set 8 were divergent. The validation accuracy of Sets 1–7 improved, to approach 1.0, while the validation accuracy of Set 8 remained at 0.5 and did not improve (dashed blue line). DenseNet121 showed lower performance in the ODIR dataset (Set 5, solid red line) but tended to improve as the learning process progressed. After 40 epochs, DenseNet121, ResNet50, and VGG19 showed similar excellent performance in Sets 2–4.

In this study, we built several CNNs to distinguish between right and left fundus photographs. We investigated whether CNNs can distinguish left and right fundus photographs even when one image is horizontally flipped. We used class activation mapping^9^ (CAM) to determine which part of the fundus image was important for discriminating the photographs (Fig. 1).

## Results

### Baseline characteristics

Medical charts of patients who visited Gyeongsang National University Changwon Hospital, South Korea (GNUCH) between February 2016 and December 2020 were reviewed retrospectively. We reviewed all fundus examinations conducted during this period and collected 101,494 fundus images. We initially included all images to reduce selection bias. The images included macula-on rhegmatogenous retinal detachment, mild vitreous hemorrhage, and images filled with gas, air, or silicone oil. We excluded 3456 images because the macula and optic disc could not be identified or because the macula was located significantly far from the center of the image. Therefore, 98,038 images were included in this study. An ophthalmologist labeled the images as being from the left or right eye. There were 48,504 images of the right fundus and 49,534 images of the left fundus. The Ocular Disease Intelligent Recognition (ODIR) dataset^10^ has 8000 images, 4000 images each from right and left eyes, and we included all these.

### Comparison of right and left fundus images (Set 1)

**Figure 2:**
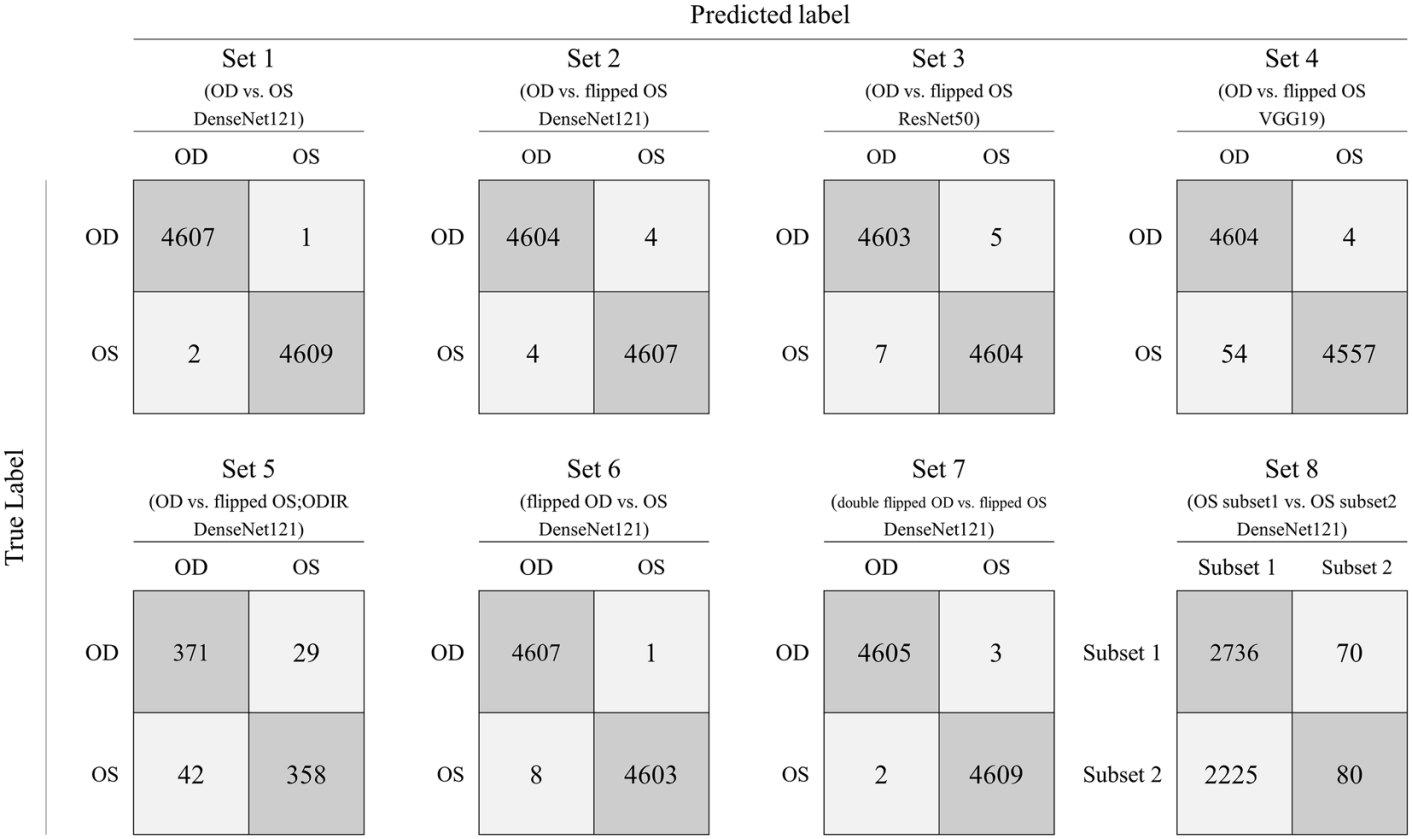
Confusion matrix for the test sets. Test Set accuracies were higher than 99% in all Sets but 5 and 8. The depth and structure of the CNN affected the accuracy slightly (Sets 2, 3, and 4; DenseNet121: 99.91%, ResNet50: 99.86%, and VGG19: 99.37%). The ODIR dataset with DenseNet121 model showed a significantly lower accuracy (91.1%, area under the curve = 0.912, p < 0.001). For Set 8, 97.1% of the images were classified as subset 1, and the test set accuracy was 55.1% (area under the curve = 0.505, p = 0.548).

**Figure 3:**
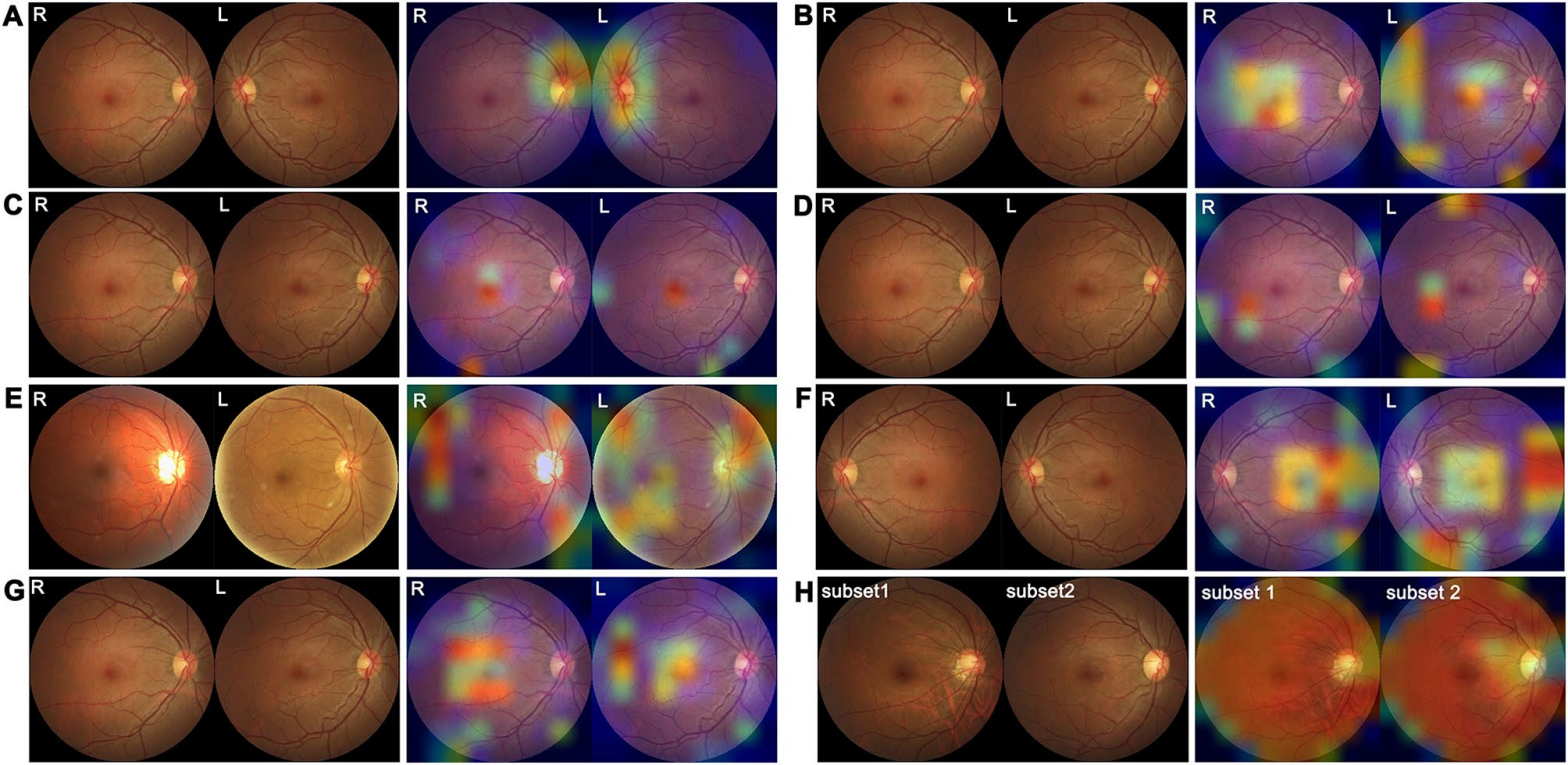
Image Sets with their corresponding class activation mapping (CAM) results. (**A**) Set 1 consisted of fundus images of right and left eyes. CAM highlighted the optic disc as the discriminative region. (**B**) Set 2 consisted of non-flipped right fundus images and flipped left fundus images. It was difficult to identify right and left images, but CAM highlighted the macula as the discriminative region. (**C,D**) The fundus photographs of Sets 3 and 4 were identical to Set 2 but used the ResNet50 and VGG19 models, respectively. CAM also highlighted the macula as the discriminative region. (**E**) Set 5 used the DenseNet121 model on the ODIR dataset. After left images were flipped, we tried to discriminate right images from left images. CAM highlighted not only the macula but also the superonasal and inferonasal retinal nerve fiber layer. (**F**) Set 6 consisted of flipped right fundus images and non-flipped left fundus images. The results were indistinguishable from those with Set 2. (**G**) Set 7 consisted of right fundus images flipped twice and left fundus images flipped once. CAM highlighted the macula as the discriminative region. (**H**) Set 8 consisted of images of only the left eye flipped once. As the entire image was highlighted in CAM, we could not specify the discriminative region.

We classified GNUCH dataset images using the DenseNet121 model, which is a CNN. The validation accuracy after the 1st epoch was 99.96%, which was extremely high (Fig. 1). After the 50th epoch, 9216 of 9219 test set images were correctly labeled by the CNNs, and the test accuracy was 99.97% (Fig. 2). CAM highlighted the optic disc as the discriminative area (Fig. 3A).

### Comparison of non-flipped right fundus images and left fundus images flipped once (Set 2)

We classified the right and horizontally flipped left fundus images using the DenseNet121 model. The numbers of images in Sets 1 and 2 were equal. The validation accuracy after the 1st epoch was 98.97% (Fig. 1). After the 50th epoch, 9211 of 9219 test set images were classified correctly. The test set accuracy was 99.91% (Fig. 2). CAM identified the macula as the discriminative area for the decisions of the CNNs (Fig. 3B).

### Comparison of DenseNet121, ResNet50, and VGG19 models on the same datasets (Sets 2, 3, and 4)

Sets 2, 3, and 4 used identical fundus images, of right and horizontally flipped left ones, but with a different CNN applied to each (DenseNet121^11^, ResNet50^12^, and VGG19^13^, respectively). The models in order of their depth, deepest first, are DenseNet121, ResNet50, and VGG19. The depth and structure of the CNN affected the learning process (Fig. 1) and test set accuracy (Fig. 2). The deeper the CNN, the faster the loss convergence. Furthermore, deeper models showed better performance (DenseNet121: 99.91%, ResNet50: 99.86%, VGG19: 99.37%).

### ODIR dataset with non-flipped right fundus images and left fundus images flipped once (Set 5)

The ODIR dataset^10^ is composed of 4000 each right and left fundus photographs. We classified right and horizontally flipped left fundus images using the DenseNet121 model. As the number of epochs increased, the CNN tended to reduce the training and validation losses. After the 50th epoch, 729 of 800 test set images were classified correctly. The test set accuracy was 91.13% (Fig. 2, area under the curve [AUC] = 0.912, *p* < 0.001).

### Comparison of flipped right fundus images and non-flipped left fundus images (Set 6)

We classified the Set 6 images using the DenseNet121 model. After the 50th epoch, 9210 of 9219 test set images were classified correctly. The test set accuracy was 99.90% (Fig. 2). CAM showed indistinguishable results from Set 2 (Fig. 3F).

### Comparison of right fundus images flipped twice and left fundus images flipped once (Set 7)

We classified the Set 7 images using the DenseNet121 model. The number of images in this set was the same as that in Set 1. The validation accuracy after the 1st epoch was 98.14%. After the 50th epoch, 9214 of the 9219 test set images were classified correctly. The test set accuracy was 99.95% (Fig. 2). CAM for Set 3 showed a pattern similar to that seen by CAM for Set 2 (Fig. 3G).

### Comparison between flipped left fundus images distributed randomly (Set 8)

We used the CNNs to classify randomly distributed flipped images of the left fundus. We randomly divided 49,534 images of the left fundus into subset 1 and subset 2 (24,268 in subset 1; 24,266 in subset 2). There was no significant decrease in training loss during training (1st epoch: 0.718; 50th epoch: 0.696; Fig. 1). The validation accuracy after the 50th epoch was 49.86%. Of the 5111 test set images, 4961 images (97.1%) were classified as subset 1 (AUC = 0.505, p = 0.548). The test set accuracy was 55.1% (Fig. 2). The entire image was highlighted by CAM so it was not possible to identify the discriminative location (Fig. 3H).

## Discussion

The fundus images of the left and right human eyes differ markedly. The macula is located in the center of the fundus image and the optic disc is located on the nasal side of the image. The major retinal arteries and veins run side-by-side into the superotemporal, inferotemporal, superonasal, and inferonasal quadrants. However, if the fundus image of one eye is horizontally flipped into a mirror image, the positions of the optic disc, macula, and retinal vessels are similar to those of the other eye. This is analogous to the image of one’s right hand in the mirror being similar to the left hand. It is thought that the left and right eyes have mirror symmetry with significant morphological differences between them.

CNNs have developed greatly in recent years. Images are processed through several hidden layers. In contrast to conventional machine learning, a CNN considers together only those pixels that are close to each other in the image^14^. CNNs have improved spatial and shape recognition, and have shown high-quality performance in several studies^15^. Fundus images are important for diagnosing retinal diseases and can even be obtained with a smartphone^16^. Many studies have evaluated the use of CNNs to classify fundus images^6^. Data augmentation increases the accuracy of classification tasks and reduces overfitting^17,18^. Image flipping, the traditional affine transformations method, is frequently used in deep learning with fundus photographs^7,8^. However, we thought that flipped fundus photographs might have distinctive features. Then the CNN might learn features from the flipped images, and the flipping method could induce undesired bias.

We investigated whether CNNs could identify fundus images as being from the right or left eye (Set 1). Because the mirror images of the two eyes are markedly different, it is easy to distinguish their fundus images. Our CNNs were able to differentiate between the left and right fundus images with 99.97% accuracy.

Next, we flipped the fundus images of the left eye horizontally using NumPy^19^ software (Set 2). The CNNs successfully identified the non-flipped images from the right eye and the flipped images from the left eye with 99.91% accuracy. This high level of accuracy exceeded our expectations because no distinct features have ever been reported that distinguish right and left fundus images. To confirm the results for Set 2, we designed Sets 3–8.

First, we tried several different deep learning models that had distinct layer depths and structures. Sets 3–5 used an identical dataset with different CNNs: DenseNet121^11^, ResNet50^12^, and VGG19^13^, respectively. As their names imply, DenseNet121, ResNet50, and VGG19 have approximately 121, 50, and 19 layers, respectively. VGG19 has a classic CNN structure and connection and was published in 2014. ResNet50 was developed to overcome the gradient vanishing problem of VGG19 and improve results from the ImageNet database. DenseNet121 was developed to modify ResNet50 by connecting densely between layers and was modified to have fewer trainable weights. As the models have distinct numbers of layers and trainable weights, it is valuable to compare them. DenseNet121 and ResNet50 showed comparable test results (99.91% and 99.87%, respectively), and ResNet50 took more epochs to reach convergence and give its final results. VGG19 showed more fluctuation in its training and validation results, and its test set accuracy (99.37%) was lower than those of DenseNet121 and ResNet50. Despite these subtle differences, all models could distinguish the right photographs from the flipped left photographs with a high accuracy (> 99%).

Sets 1–4 consisted of images from the GNUCH dataset taken using a single device. We compared the ODIR dataset results of Set 5 to the GNUCH dataset results of Sets 1–4. The ODIR dataset is a “real-life” Set of information collected from patients at different hospitals in China. In these institutions, fundus images were captured by various cameras available commercially, such as by manufacturers Canon, Zeiss, and Kowa, resulting in varied image resolutions^10^. Set 5 showed a lower accuracy but significant results (91.1%, AUC = 0.912, *p* < 0.001). Therefore, we can presume that similar results would be achieved with other cameras and at other institutions. The interocular asymmetry that we found might be a universal result. The main reason for lower ODIR dataset accuracy than the GNUCH dataset is that the former consists of heterogeneous images. The GNUCH dataset consists only of fundus photographs in which both the macular and optic discs can be recognized. However, the ODIR dataset included 448 fundus photographs in which these could not be recognized; these were images with severe opacity, vitreous hemorrhage, or images outside the posterior pole. Also, 99 images were distorted, and 4 were not fundus photographs. Thus, the number of images meeting the GNUCH dataset inclusion criteria was 7449 (93.1%).

Our next step was to verify the flip function in the NumPy^19^ package. The flip function simply inverts the image based on one axis and no operations are performed on the numbers constituting the matrix. Because the NumPy package could have an unknown error in the internal code, we decided to exclude the possibility of any image distortions that were not recognized by the human eye. We flipped the fundus images of both the left and right eyes to ensure that any image distortion would affect all images. Therefore, we prepared Set 6 by flipping the right fundus photographs, and Set 7 by flipping the right fundus images twice and the left fundus images once. The results for Sets 6 and 7 showed 99.90% and 99.95% accuracy, respectively, which was similar to that seen for Set 2.

Our next step was to confirm that the classification by the CNNs was based on identifying differences between the left and right eyes. If our results were due to unrecognized overfitting and not because of real differences between the left and right fundus images, a similar high level of accuracy would have been seen if the CNNs had been trained with any dataset. Conversely, if the CNNs found real differences between right and left fundus images, the CNNs trained with fundus images of only one eye would fail to discriminate between these. In this case, the accuracy would have been almost 50% because there were two classes; the accuracy of Set 8 was 55.1%, which was very close to 50% (AUC = 0.505, *p* = 0.548). The CAM pattern for Set 8 was also very different from that seen for Set 2 (Fig. 3). These findings support that the result for Set 2 was indeed due to the CNNs identifying a differentiating feature between the right and left eyes.

We cannot explain these results of CNNs. However, CAM was able to identify which part of the image was more important for image classification using CNNs. In Set 1, the optic disc had a high weight on CAM. The optic disc is therefore the part with the greatest difference in the fundus images of the two eyes. In this scenario, CNNs and CAM are similar to humans, because we too distinguish left and right fundus images by looking at the position of the optic disc. By CAM for Sets 2–7, the macula had the highest weight, and the peripapillary area was also highlighted in Set 5. We verified this by creating models through several iterations. With each iteration, the CAM patterns changed slightly, but the point with the highest weight was consistently the macula. There are several conclusions to be drawn from these results. First, the JPG file format, NumPy, and OpenCV were not responsible for the CAM pattern of the macula because they would have affected the entire image equally. Second, in fundus photography, blurred images are usually caused by small pupil size and are more pronounced around the edges of the images, so they will not create a localized high weight on the macula in the CAM pattern. Third, the optic disc is not important when identifying flipped fundus images.

We found an asymmetry between the maculae of the left and right eyes. Cameron et al.^20^ reviewed many studies about the asymmetry of interocular visual systems, and they concluded that it was obvious, but they did not identify any specific components. A possible explanation for such asymmetry could lie in the retinal nerve fiber layer (RNFL). Wagner et al.^21^ reported that the angles between the upper and lower maxima of peripheral RNFL thickness areas in right eyes were higher than in left eyes. RNFL asymmetry may be influenced by interocular differences in the locations of the superotemporal retinal artery and vein^22^. CAM of the ODIR dataset (Set 5) highlighted the peripapillary RNFL area, as did the GNUCH dataset under DenseNet121 (Sets 2, 6, and 7) but to a more limited extent. Another explanation of interocular asymmetry is retinal vascular asymmetry: Leung et al.^23^ revealed that the mean central retinal arteriolar equivalent of right eyes was 3.14 µm larger than that of left eyes. Although the naked eye cannot detect these subtle interocular differences in RNFL and retinal vessels, CNNs may be able to find them.

The macula is the most important area for central vision, and it appears as a slight depression in the retina. As shown in Fig. 3, the maculae of both eyes appear to have mirror symmetry. However, the macula has an unknown distinguishing feature that allows left and right fundus images to be discriminated with > 99.9% accuracy. It is not possible to reveal the classification process of the CNNs, but we can make two assumptions. First, the degree and angle of the depression of the macula of the two eyes differ, which may affect light reflection. Differences in the depth of depression of the macula may be identified by optical coherence tomography. Second, the size, shape, and color of the macula may differ between the two eyes. However, such differences have never been reported in the literature. It was a limitation of this study that we did not use CNNs to distinguish between images and therefore had to rely on an indirect method, CAM. Further study on this topic is needed.

In conclusion, until now it was thought that fundus images of both eyes are mirror symmetric. However, we found asymmetry in the macula of the two eyes that allowed CNNs to discriminate between them with an accuracy > 99%. To the best of our knowledge, this is the first study on differences in the maculae of both eyes. CNNs can be used to discriminate fundus images into left and right eyes. In addition, macular asymmetry could introduce bias in CNNs using fundus images. This bias may affect the results of machine learning studies on diseases that involve the macula, such as age-related macular degeneration and diabetic macular edema.

## Methods

### Study design

The protocol of this retrospective study was approved by the institutional review board of GNUCH. The procedures used in this study followed the principles of the Declaration of Helsinki. The requirement for obtaining informed patient consent was waived by the institutional review board (GNUCH 2021-05-007) due to the retrospective nature of the study.

### Image acquisition protocol

An expert examiner photographed the fundus with a digital retinal camera (CR-2; Canon, Tokyo, Japan). The images were stored in a picture archiving and communication system. We accessed the images using automated programs written in AutoIt and saved them in JPG file format in the GNUCH dataset. We used the ODIR dataset^10^ to validate the GNUCH dataset. The ODIR dataset is open access and was collected by Shanggong Medical Technology in China.

### Pre-processing step

**Figure 4:**
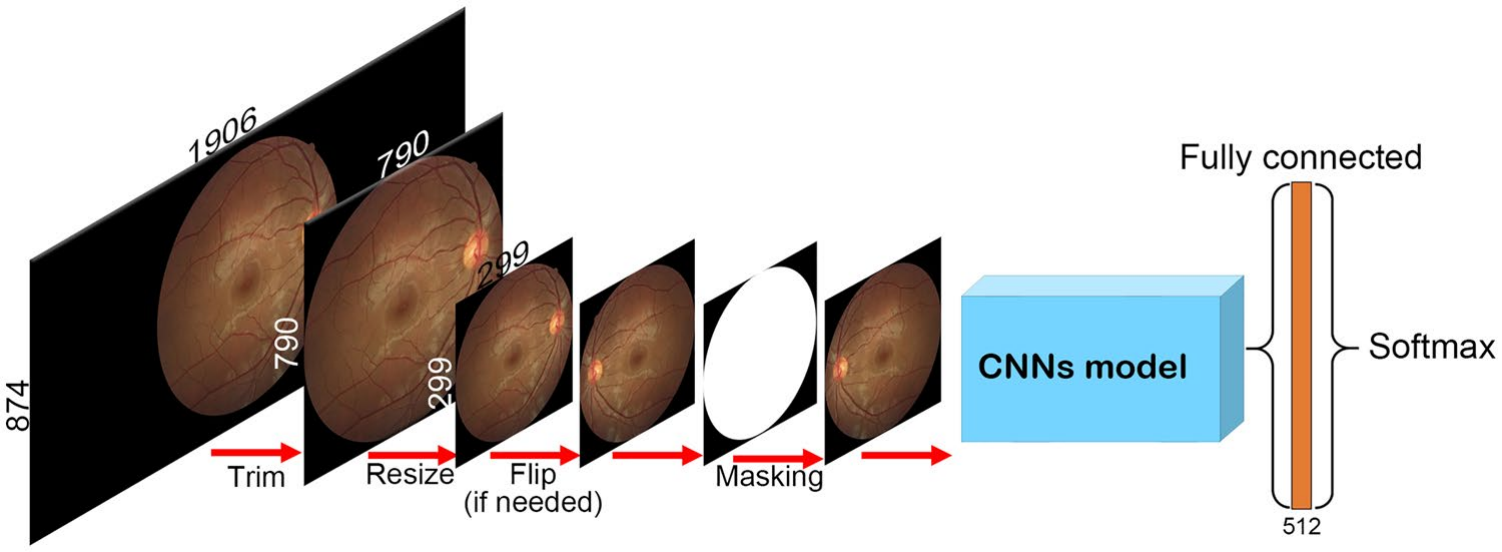
Diagram of the research process. The edges of the images were trimmed to decrease the image size from 1906 × 874 to 790 × 790 pixels. The images were then re-sized to 299 × 299 pixels. If needed, the images were flipped horizontally. A round mask was used to create identically shaped images. The processed images were loaded into CNNs connected to the fully connected and softmax layers.

We did not normalize the color in, or remove noise from, the images. We processed the images using Python software packages, especially OpenCV and NumPy (Fig. 4). The edges of the images in the GNUCH dataset were trimmed to decrease the image size from 1906 × 874 pixels to 790 × 790 pixels using NumPy. We also trimmed any blank area of the ODIR dataset images. The images were then re-sized to 299 × 299 pixels using OpenCV. If needed, NumPy was used to flip the images horizontally once or twice. The images were further trimmed so that they were all circular.

### Convolutional neural networks

To improve accuracy, we used a pre-trained model to transfer learning^24–27^. This model had been trained on a large dataset for a previous study. A pre-trained model contains the weights and biases representing features of the dataset on which it was trained. These features are transferable to different data, such as images of the eye^26^. We constructed our model using DenseNet121^11^, ResNet50^12^, and VGG19^13^ of the Keras software package. CNNs’ output was connected to the fully-connected and the softmax layers. We used categorical cross-entropy loss functions and Adaptive Moment Estimation as the gradient descent optimization algorithm.

### Image set

We prepared eight image Sets (Fig. 3). Sets 1–4 and 6–8 consisted of the GNUCH dataset, and Set 5 consisted of the ODIR dataset. The number of images of Sets 1–4, 6, and 7 were identical, consisting of training, validation, and test sets of 82,967, 4852, and 9219 images, respectively. Set 8 consisted of only left fundus images; thus, it had about half the number of images: 41,496, 2427, and 4611 images, respectively. Set 5 consisted of 6400, 800, and 800 images, respectively. We sampled images for the validation and test sets with the random.sample function in Python.

Set 1 consisted of GNUCH dataset images of right and left eyes without any transformation (Fig. 3A). Sets 2–4 consisted of GNUCH dataset images of non-flipped right fundus images and horizontally flipped left fundus images (Fig. 3B–D). Set 5 consisted of ODIR dataset images of non-flipped right fundus images and flipped left fundus images (Fig. 3E). Set 6 consisted of GNUCH dataset images of horizontally flipped right fundus images and non-flipped left fundus images (Fig. 3F). Set 7 consisted of right fundus images flipped horizontally twice and left fundus images flipped horizontally once (Fig. 3G). The images in Sets 1–7 were labeled as showing right and left eyes. Set 8 consisted of images of only the left eye flipped once. The images in Set 8 were randomly divided into subset 1 or subset 2 (Fig. 3H).

### Class activation mapping

We used CAM^9^ to understand how the CNNs worked because it is a technique used to visualize decisions made by CNNs. CAM heatmaps identify areas used by CNNs to make a decision, with redder areas carrying more weight in a heatmap, indicating a more important part of CNN class discrimination. By using CAM, we could identify the locations that carried more weight in the final convolutional and classification layers.

### Software

Python version 3.7.9 was used for this study. The CNNs consisted of TensorFlow 2.4.1, Keras 2.4.3, OpenCV 4.5.1.48, and NumPy^19^ 1.19.5. The performance of each CNN was evaluated by calculating the accuracy of the test set. The CPU used to train the CNNs was an Intel^®^ Core™ i9-10980XE, and the GPU was a GeForce RTX 3090 Graphics Card. We analyzed the results of the test set using SPSS statistical software version 24.0 (SPSS, Chicago, IL, USA) for Microsoft Windows. Receiver operating characteristic curves and their AUCs were computed to evaluate predictivity. Statistical significance was Set at a value of p < 0.05.

## Data Availability

Data supporting the findings of the current study are available from the corresponding author upon reasonable request.
