# Author Correction: Asymmetry between right and left fundus images identified using convolutional neural networks

**DOI:** 10.1038/s41598-022-10443-1

**Published:** 2022-04-13

**Authors:** Tae Seen Kang, Bum Jun Kim, Ki Yup Nam, Seongjin Lee, Kyonghoon Kim, Woong-sub Lee, Jinhyun Kim, Yong Seop Han

**Affiliations:** 1 Department of Ophthalmology, Gyeongsang National University Changwon Hospital, #11 Samjeongja-ro, Seongsan-gu, Changwon, 51472 Republic of Korea; 2 Department of Ophthalmology, Chungnam National University Sejong Hospital, Sejong, Republic of Korea; 3 Department of AI Convergence Engineering, Gyeongsang National University, Jinju, Republic of Korea; 4 School of Computer Science & Engineering, Kyungpook National University, Daegu, Republic of Korea; 5 Department of Information and Communication Engineering, Gyeongsang National University, Tongyeong, Republic of Korea; 6 Department of Ophthalmology, Institute of Health Sciences, Gyeongsang National University College of Medicine, Jinju, Republic of Korea

Correction to: *Scientific Reports* 10.1038/s41598-021-04323-3, published online 27 January 2022

The original version of this Article contained an error in the spelling of the author Yong Seop Han, which was incorrectly given as Yong Soep Han.

This error has now been corrected in the original Article.

